# Ecological meta‐analyses often produce unwarranted results

**DOI:** 10.1002/ecy.70269

**Published:** 2025-12-08

**Authors:** Scott D. Peacor, Chao Song, James R. Bence, Amy A. Briggs, Elizabeth A. Hamman, Craig W. Osenberg

**Affiliations:** ^1^ Department of Fisheries and Wildlife Michigan State University East Lansing Michigan USA; ^2^ State Key Laboratory of Herbage Improvement and Grassland Agro‐ecosystems College of Ecology, Lanzhou University Lanzhou Gansu People's Republic of China; ^3^ Department of Evolution and Ecology UC Davis Davis California USA; ^4^ Department of Biology St. Mary's College of Maryland St. Mary's City Maryland USA; ^5^ Odum School of Ecology University of Georgia Athens Georgia USA

**Keywords:** hierarchical model, meta‐analysis, nonindependence, pseudoreplication, random effect

## Abstract

Meta‐analysis (MA), a powerful tool for synthesizing reported results, is influential in ecology. While ecologists have long been well‐informed on the potential problems associated with nonindependence in experimental work (e.g., pseudoreplication), they have, until recently, largely neglected this issue in MA. However, results used in MAs are likely much more similar when they come from the same locality, system, or laboratory. A simple and common form of nonindependence in MA arises when multiple data points, that is, observed effect sizes, come from the same paper. We obtained original data from 20 published MAs, reconstructed the published analyses, and then, for 14 that had not accounted for a paper effect, used three approaches to evaluate whether within‐paper nonindependence was a problem. First, we found that “nonsense” explanatory variables added to the original analyses were statistically significant (*p* < 0.05) far more often than the expected 5% (25%–50% for four nonsense variables). For example, the number of vowels in the first author's name had a significant effect 50% of the time. Second, we found that an added dummy variable, which was randomly assigned at one of two levels, was statistically significant an average of 38% of the time, far exceeding the expected 5%. Even after including a random paper effect in the analyses, there was still an excess of significant results, suggesting that the within‐paper nonindependence was more complex than modeled with the random paper effect. Third, we repeated the original MAs that did not include random paper effects (*n* = 14 MAs) but added a random paper effect to each revised analysis. In 12 out of the 14 MAs, an added random effect was statistically significant (indicating group nonindependence that was not accounted for in the original analyses), and often the original inferences were substantially altered. Further, incorporating random paper effects was not a sufficient solution to nonindependence. Thus, problems resulting from nonindependence are often substantial, and accounting for the problem will likely require careful consideration of the details of the potential dependence among observed effect sizes. MAs that do not properly account for this problem may reach unwarranted conclusions.

## INTRODUCTION

Given its promise to increase the rigor of data synthesis, the use of meta‐analysis (MA) has grown in influence in science (Gurevitch et al., 2018; Hoffmann et al., 2021; Vrieze, 2018), with the number of published ecological MAs increasing rapidly since their early use in the 1990s (Gurevitch et al., 2018; Lortie, 2014; Vrieze, 2018). Many papers published in major ecological journals cite multiple MAs both as background and when relating new results to existing knowledge. As a result, ecological MAs influence our understanding of diverse topics including the effects of global climate change on disease risk (Mahon et al., 2024), invasive ants on animal diversity (Tercel et al., 2023), pollination on agricultural services (Gazzea et al., 2023), and marine protected areas on biodiversity (Hollitzer et al., 2023). Thus, much of ecology, as well as the application of ecology to management and policy, is guided by syntheses informed by MA. Clearly, any method with such widespread influence ought to be based on sound and well‐tested methodology. Nevertheless, problems with the application of MA in ecology have been recognized (Chamberlain et al., 2012; Nakagawa et al., 2023; Osenberg et al., 1999; Pappalardo et al., 2023; Yang et al., 2023).

One potential challenge with the application of MA is nonindependence in which a MA uses multiple data points (i.e., observed effect sizes) from the same “group”—paper, laboratory, taxon, or site, which might not be independent (Koricheva & Gurevitch, 2014; Nakagawa et al., 2017; Noble et al., 2017). If nonindependence within groups is ignored (a form of pseudoreplication: Hurlbert, 1984), it can lead to poor estimates of average effect sizes, undermine the validity of inferences, and lead to unwarranted conclusions (Nakagawa et al., 2022; Noble et al., 2017; Song et al., 2020, 2022). Indeed, ecologists are keenly aware of the potential problem of nonindependence in experiments. Generally, independence in experiments is achieved by randomly assigning experimental units to treatments. However, despite ecologists' appreciation of nonindependence in experimental ecology (e.g., Hurlbert, 1984 has been cited >10,000 times), attention to nonindependence in early ecological MAs was largely ignored. Indeed, until recently, commonly used MA software in ecology (e.g., MetaWin [Rosenberg et al., 2000] which is still in use, but less commonly today) could not accommodate random group effects or otherwise account for nonindependence. This left some meta‐analysts reaching for ad hoc solutions that have been shown to be insufficient (Song et al., 2020).

Over the past two decades, publications in the ecological literature have introduced methods to account for nonindependence, giving the impression that ecologists are increasingly accounting for this issue. Phylogenetic nonindependence was one of the first forms that ecologists attempted to address, and this early work highlighted the potential problems associated with unaccounted‐for nonindependence (e.g., Chamberlain et al., 2012; Hoeksema et al., 2010; Lajeunesse et al., 2013; Morales & Traveset, 2009). Subsequently, the development of additional methodologies and software was used to address other forms of nonindependence (Hedges et al., 2010; Nakagawa et al., 2022; Pustejovsky & Tipton, 2018; Viechtbauer, 2010; Yang et al., 2024). Since simulations have demonstrated that accounting for nonindependence caused by group effects, when they exist, can lead to more valid inferences (Nakagawa et al., 2022; Song et al., 2020), the increased implementation of methods to account for nonindependence is a positive step. However, the uptake of these methods has been less than ideal. The most complete and recent review of this issue (Pappalardo et al., 2023) reported on two types of potential nonindependence that could arise when extracting >1 effect size from a primary publication: (1) within‐study: the same data were used for calculating more than one observed effect size, and (2) study‐level: different data were used although the effects might still be nonindependent due to other similarities (e.g., sharing common methodological biases or coming from the same geographic locality). They found that 65% of the 96 ecological MAs in their dataset acknowledged and attempted to address the within‐study source (generally by averaging or subsampling to use just one observed effect that used common data), but only 14% acknowledged and addressed the study‐level source of nonindependence. These sources of nonindependence are particularly problematic, as they may arise whenever the number of observed effect sizes exceeds the number of source papers, which Pappalardo et al. (2023) found to occur in 95 out of 96MAs, with many MAs reporting more than five times more effect sizes than papers.

Despite the recognition of the problem posed by nonindependence, we still do not know the magnitude of the problem in ecological MAs. Is nonindependence little more than an esoteric statistical issue with little real consequence to the results of an MA? Our own discussions with meta‐analysts in recent years have suggested that many view the problem in this way, implicitly assuming that group effects are negligible or that the occurrence of multiple effects within groups is uncommon and not influential (and thus can be ignored without compromising the conclusions). Further substantiating this observation, MAs performed without accounting for group effects (or without sensitivity analyses of the potential group effects) continue to be published, and older published MAs that did not account for group effects continue to be highly cited. Thus, ecologists as a community appear to have faith in the inferences drawn from MAs that overlook nonindependence. Clearly, we desire rigorous approaches, but rigor and exactitude are often traded off with the expediency of not accounting for nonindependence.

Here, we examine the consequences of that trade‐off and evaluate the magnitude of the error that results from ignoring nonindependence. We focus on a particularly common type of group effect: nonindependence of observed effect sizes from the same paper (Song et al., 2020). Within‐paper nonindependence could arise from many factors (Noble et al., 2017), which could include random factors that influence actual (“true”) effect sizes, as well as factors that only influence the observed effect size (herein we use *actual effect size*, and for short, *effect size*, to refer to the effect that would be seen if infinite data were available, as distinct from the *observed effect size* which incorporates observation error, reflecting that the true effect size is not directly observed). For example, when a paper yields >1 observed effect size, those often are for the same species (or closely related species) or from the same locale (or a limited geographic region). Thus, they would tend to have correlated true effect sizes. Furthermore, because the same author(s) often designed and executed the experiments, the effects likely were influenced by methodological similarities. Observed effect sizes might also have come from the same experiment but at different levels of another treatment (e.g., in cross‐factored designs), a case where random influences on true effect sizes and the observation errors might both contribute to nonindependence. Multiple observed effect sizes might also have been taken from the same experiment, but represent different response metrics (e.g., biomass or growth rate to represent productivity). If these responses are measured from the same set of organisms, they might have shared a common control (and thus some of the same data might have been used in the calculation of the observed effect sizes). Regardless of whether factors influenced the actual effect size, or just the observation process, or both, in all these cases, we would expect that observed effect sizes will be more similar when taken from the same paper. Therefore, we focus on paper effects rather than other groupings (e.g., geographic region, phylogeny, effects from the same laboratory or closely related laboratory) because it is likely a very common source of nonindependence, it is a tractable grouping, and it is the simplest grouping for us to examine. Because within‐paper effects are just one source of nonindependence, identifying the magnitude of paper effects is a conservative evaluation of the overall problems associated with nonindependence—problems are likely to be even more profound than what we identify. Thus, while we focus on a particular type of nonindependence, our goal is to examine the influence of the potential effects of nonindependence generally, and we use within‐paper nonindependence as one example of a larger issue.

## METHODS

### Overview

We used three approaches to examine and elucidate the influence of within‐paper nonindependence. All three approaches were based on published MAs, using the original data, and applying the same analytical methods as the authors, albeit with specified modifications germane to our approach. Using published data was critical to our message, as the nature of the data will influence the effect of within‐paper nonindependence (e.g., Song et al., 2020), and our goal was to evaluate the error in typical ecological analyses. Being able to repeat the original analyses also was important, as it conserved the original approach of the authors.

In our first approach, we tested if nonsense binary categorical variables (e.g., 0 or 1) assigned to source papers (e.g., based on two groupings of the number of vowels in the first author's given name) yielded a significant effect. We separately tested four nonsense categorical variables. In our second approach, we tested the significance of the effect of a binary categorical variable, whose level was assigned randomly to all observed effect sizes in a source paper, thus guaranteeing that its level was unrelated to the actual effect size. This mimics the reality that most observations from the same source paper end up in the same category defined for a MA. While the second approach is a formal analysis, we include the first approach (which is not) because our experience when communicating our results is that the first approach is more intuitive and more attention‐grabbing, which, given the purpose of our overall exercise to bring attention to the issue, is desirable. For both these cases, there should be no effect of the nonsense or randomly assigned binary variables, but we hypothesized that nonindependence could cause too many false significant results (more than the nominal 5% for a 0.05 level test). We suspected this because multiple effect sizes from the same source paper would tend to be similar, and these similar values would all end up in the same level of the binary variables. This would lead to an underestimation of residual error variance, and thus too many significant results. We repeated the first two approaches after incorporating a simple random paper effect to evaluate whether this widely used approach was sufficient to resolve any issues of within‐paper nonindependence identified by the first two approaches.

In our third approach, we added a random paper effect to the original statistical model to see how that altered the significance (*p*‐value) of the moderator categorical variable evaluated in the original MA, and whether the random paper effect was significant. We included this third approach because it is a widely used way ecological MAs attempt to account for nonindependence within papers. Although there are sophisticated and targeted model‐based ways to address some of the various forms of within‐paper nonindependence, they require specification of variance–covariance relationships among the effect sizes for each original paper, which are substantially more challenging to implement.

### Data collection method

Our goal was to acquire 20 MA papers representative of those used in the broader ecological literature, and to use those papers to quantify the level of error associated with ignoring within‐paper nonindependence. We searched Web of Science's “All Databases” for papers with the topic “meta‐analysis” or “metaanalysis” that were published in 2018, and that were in the following research areas: biodiversity conservation, parasitology, evolutionary biology, plant sciences, forestry, marine biology, freshwater biology, environmental sciences, ecology, or zoology. We further refined the search to include only “article” or “review” document types. This search returned 3277 papers from journals with a broad focus on basic ecology questions, such as *Ecology Letters* and system‐specific journals, such as the *Canadian Journal of Soil Science*. We then examined the abstracts of the papers returned from this search and excluded papers that did not perform a MA or did not fall within the fields of ecology or evolution. Therefore, for example, we included MAs investigating the effects of biocontrol in agricultural systems or the responses of microbial communities to an intervention like CO_2_ enrichment, but we excluded MAs of medical trials of drug effects on gut microbiomes/parasite loads and agricultural studies that only measured the effect of an agricultural technique on crop yield. In the second screening step, we chose papers in chronological order, examined the full paper, and continued until we obtained 20 papers that fit all of the following criteria: (1) met our previous criteria; (2) weighted observed effect sizes (i.e., by inverse variance, sample size, or other measure of study quality); (3) explicitly tested for differences in mean effect sizes among multiple groups (i.e., evaluated if heterogeneity in effects was explained by at least one categorical moderator variable); (4) provided data necessary to reconstruct the analyses (either via an online supplement or in response to our request for the data); and (5) able to approximately reconstruct the statistical results reported in each paper or (in one case) ascertain through interactions with authors that our analysis was what the authors had intended to implement, but was not what they had actually conducted. When there was more than one analysis of moderators from each paper, we used the one that yielded the lowest *p*‐value for the test of the moderator. While this approach will select for lower *p*‐values, it nevertheless led to a large range from *p* < 0.0001 to *p* = 0.91 and tended to focus on the effects that were most pronounced and thus influential. About half of the MAs provided the data they used as supplementary material. For those that did not, we requested the data from the authors to avoid any bias due to potential differences between publications that provided data and those that did not. In all but two cases, authors provided the data. In all, our approach required screening abstracts of 1700 papers, then examining 165 full papers that passed the first screening step. A PRISMA diagram is provided in Appendix S1: Figure S1.

Of the 20 MAs that met our criteria, 5 included a random effect for paper in the original analysis. Our original intention to use the data from papers that included a random effect of paper and then remove that effect from the original analysis proved too difficult: for example, due to the difficulty in interpreting the methodology used. Therefore, we used papers that did *not* include a random paper effect. One of the 15 MAs that did not include a paper effect summarized data for the same sites over multiple papers to calculate individual observed effect sizes, thus making it impossible to connect an observed effect size to a single paper. Analyses and results subsequently reported in the main text of the paper are based on the 14 remaining MAs.

### Effects of nonsense variables

We evaluated the potential problem posed by within‐paper nonindependence by examining the influence of nonsense variables that used the same data and statistical methodology as the published MAs. The methodological agreement was assured either by using the code provided in the original paper (and associated archives) or through helpful discussion with the authors of the MA until we could reconstruct their original results (in one exception neither us nor the authors could reproduce the original analysis, but the authors agreed that our analysis was what they had intended). We divided the observed effect sizes (for each of the 14 MAs) into two groups according to easily generated “nonsense” variables tied to the source paper from which they came. This assigning of all the observed effect sizes in a paper to a single nonsense variable mimicked the common situation where all or most observed effect sizes within the same paper end up being assigned the same level of a moderator (i.e., a fixed effect) in the MA. The nonsense variables were chosen so that there would be no plausible connection between their values and the effect size. We chose four nonsense variables, rather than just one, as a check that results did not depend on “bad luck” for the single choice (i.e., where the nonsense variable was associated with a variable that had an effect, even though we cannot envision how this association might arise). The four nonsense categorical variables (each of which took two values) were as follows: the parity (i.e., even or odd) of the publication year, the parity of the number of letters in the first author's last name, the first initial of the first author (whether it was in the first, or second, half of the alphabet), and the percentage of vowels in the author's last name (less or more than 33%). For each nonsense variable separately, we then added it to the original statistical model as an additional fixed effect, thus obtaining a set of 14 *p*‐values for the effects of each nonsense variable. If a nonsense variable had no effect and the statistical model is correct, the resulting *p*‐values should be drawn from a uniform distribution (from 0 to 1). For each nonsense variable, we tested for departures of the *p*‐values from the expected uniform distribution using a Kolmogorov–Smirnov (KS) test.

We repeated this analysis using a model that also included a random paper effect to determine whether this common approach to address nonindependence fully mitigated any issues with statistical inferences. That is, for each of the four nonsense variables, for each of the MAs, we included a random paper effect as well as the nonsense variable, assumed to be drawn independently from a normal distribution with a mean of zero. If an excess of low *p*‐values for the nonsense variables were fully alleviated by adding a random paper effect (e.g., the proportion of significant nonsense variable effects should have been reduced to about 0.05), this would suggest that including a random paper effect was an adequate solution for within‐paper nonindependence.

### Effects of a random dummy variable

We applied a resampling procedure to our compiled set of 14 MAs that, like the nonsense variable analysis, used the same data and same methodology as the published MA studies. During each iteration of our resampling, for each of the 14 MAs included in that resample, we added a dummy variable (i.e., a categorical variable with two levels) to the original statistical model, with level assigned randomly to a group (so all observed effect sizes taken from the same group had the same level). These groups were defined by the combination of paper and the level of the focal categorical factor (moderator) being analyzed in the original MA. Most of the MAs (12 out of 14, Table 1) had just one categorical moderator. For the two MAs with more than one focal moderator, we selected the factor with the lowest *p*‐value in the original analysis. In most cases, these groups were equivalent to paper because all the observed effect sizes from a source paper fell in the same level of the categorical variables. In a few cases, a source paper yielded observed effect sizes in more than one category of the moderator. Because we assigned the dummy variable to groups randomly, the null hypothesis of no effect of this new “moderator” was true.

**TABLE 1:** Properties of the 20 meta‐analyses (MAs) initially identified.

Paper ID	RPE in original analysis	No. papers	No. studies	Average no. studies per paper	Maximum no. studies from a paper	No. moderators
53	No	38	215	5.7	36	1
2	No	47	95	2.0	12	1
82	No	32	106	3.3	6	1
120	No	20	134	6.7	16	1
511	No	35	229	6.5	32	1
695	No	99	353	3.6	31	1
759	No	26	92	3.5	16	1
1365	No	46	811	17	61	3
1307	No	65	199	3.1	12	1
1180	No	60	162	2.7	10	1
952	No	31	325	10	72	1
1185	No	69	415	6.0	28	2
674	No	17	36	2.1	10	1
639	No	24	202	8.42	84	1
585	No^a^	8	31	NA	NA	1
272	Yes	96	294	3.1	16	2
282	Yes	108	774	7.2	48	3
303	Yes	27	98	3.6	14	1
628	Yes	6	38	6.33	13	1
424	Yes	7	18	2.6	6	1

*Note:* Paper ID can be used to find the original MA publication (see Appendix S1: Table S1). “No. papers” is the number of papers and studies (i.e., individual observed effect sizes) used in the original MA. “No. moderators” is the number of variables influencing effect size in the MA model.

*Abbreviations*: RPE, random paper effect. NA, not applicable.

^a^ For Study 585, the “No” entry for “RPE in original analysis” indicates that the original analysis could not be modified to include a paper effect because the observed effect sizes used in the analysis were averages combined across source papers.

In our resampling procedure, we repeatedly took bootstrap samples (3000 iterations). Each bootstrap sample included 14 MAs chosen randomly (with replacement) from the 14 MAs we compiled. This resampling procedure accounted for random variation among the MAs and treated the compiled set of 14 MAs as representative of a larger population about which we wished to make inferences. Each bootstrap sample provided 14 *p*‐values associated with the null hypothesis that the two levels of the dummy variable had the same mean effect size. As noted above for nonsense variables, the resulting *p*‐values should be drawn from a uniform distribution (from 0 to 1), and we tested for such departures for each bootstrap sample using a KS test. If each set of the 14 *p*‐values conforms to a uniform distribution from 0 to 1, we expect 5% of the KS tests to be significant at the 5% level. We thus examined the proportion of significant KS tests to evaluate the overall evidence for nonindependence. We constructed the CIs of the proportion of significant KS tests based on a normal approximation to the binomial distribution (i.e., *v* = *p*(1 − *p*)/3000). The extent to which the mean proportion exceeded 0.05 quantifies the extent to which Type I errors are inflated across the original MAs. As with the nonsense variables, we then assessed if any problems were alleviated by incorporating a random paper effect.

We further examined two characteristics of the MAs that we expected might influence the results. First, we expected that papers with lower reported *p*‐values (as reported by the study authors) might produce a higher proportion of significant effects for the dummy variable, because strong within‐paper nonindependence, if not accounted for, contributes to the significance in the original paper. Second, we expected that MAs with more studies (i.e., observed effect sizes) per paper would have a higher proportion of erroneously significant effects of the dummy variable because, all else equal, we expected the departure from independence would be stronger when more of them came from the same paper.

We then evaluated if the problem of nonindependence in the original MAs was alleviated by accounting for within‐paper nonindependence by adding a random paper effect to the statistical model used to test the random dummy variables. That is, using the same 3000 MA datasets created by randomization we now applied a statistical model that added a random paper effect as well as the dummy variable, similar to what we did when adding a random paper effect to the analyses of nonsense variables, and for the same reasons.

The randomization approach we used to examine nonindependence may seem more complicated than necessary, and for completeness we explain here the evolution of our approach that clarifies the need for a complex approach. The complex approach evolved from simpler approaches we considered, but later found problematic. Originally, we tried a procedure in which we randomly assigned each paper to a level of the dummy variable, and tested for an effect of the dummy variable. We discovered that this seemingly appealing procedure led to artifactual nonindependence because “paper” was often conflated with factors included in the original MAs. We also initially explored an approach focused on individual MAs and the proportion of times the dummy variable was significant for that MA. However, in trial simulations we found that this procedure would produce average *p*‐values significantly above or below 0.05 for specific MAs, even without nonindependence or other violations of the model. The problem was that the null distributions for statistical tests apply to repeated sampling. A single given set of data will not be perfectly representative of the underlying distributions, and shuffling the samples around will not mimic repeated new sampling, especially for a MA with few studies. Thus, we had to develop the more complex simulation approach presented here.

### Effect of the random paper effect in the original analyses

To further elucidate the influence of ignoring within‐paper nonindependence, we modified the original analyses of the 14 published MAs by including a random paper effect to account for within‐paper nonindependence of studies. This is the same model we used above, except that no random or nonsense variable was used. We evaluated the degree to which including the paper effect influenced *p*‐values by looking at the ratio of the *p*‐value with and without the random paper effect. In some cases, we could not exactly reproduce the *p*‐value reported in the original paper, and in one case there was a substantial departure (Table 2). However, based on discussions with the authors, the analytic approach we took closely approximates what they did (or in one case intended to do).

**TABLE 2:** Summary analysis results for the 14 meta‐analyses (MAs) that did not originally include random paper effects (RPEs) and were used in our analyses.

Paper ID	*p* reported	*p* reproduced	*p* with added RPE	*p* for test of RPE	Prop. sig.	Prop. sig with RPE
53	<0.0001	1.9E‐21	1.4E‐06	<1E‐33	0.707	0.082
2	<0.0001	0.66	0.67	0.77	0.011	0.015
82	<0.0001	<1E‐33	0.0032	3.6E‐08	0.805	0.066
120	<0.001	2.3E‐28	2.6E‐18	2.2E‐10	0.644	0.165
511	<0.0001	<1E‐33	<1E‐33	<1E‐33	0.997	0.783
695	< 0.0001	6.2E‐50	0.0018	<1E‐33	0.493	0.076
759	0.02	0.022	0.033	0.00043	0.069	0.030
1365	< 0.001	0.0056	0.20	<1E‐33	0.548	0.106
1307	<0.001	0.044	0.070	0.0055	0.075	0.125
1180	0.007	0.0072	0.31	0.00042	0.082	0.0594
952	<0.001	2.3E‐33	6.1E‐12	0.0013	0.202	0.056
1185	<0.001	1.52E‐23	4.6E‐18	5.1E‐15	0.352	0.052
674	0.0034	0.0034	0.89	8.5E‐06	0.231	0.097
639	0.91	0.89	0.91	0.095	0.139	0.049
585	<0.01	2.53E‐30	NA	NA	NA	NA
272	<0.001	0.0043	NA	NA	NA	NA
282	0.04	0.041	NA	NA	NA	NA
303	0.023	0.023	NA	NA	NA	NA
628	NR	0.16	NA	NA	NA	NA
424	0.61	0.61	NA	NA	NA	NA

*Note*: Paper ID can be used to find the original MA publication (see Appendix S1: Table S1). The “*p* reported” is the statistical significance of the focal moderator (see text) as reported in the original MA publication, and “*p* reproduced” is the value for that test reproduced in our analyses (see text regarding differences). The “*p* with added RPE” is the *p*‐value for the test of the effect of the focal moderator after adding a RPE to a model that originally did not have one. The “*p*‐value for RPE” is the result of a likelihood ratio test for nonzero among paper variance, a test known to be conservative (i.e., tending to reject the null hypothesis less than at the stated level when there is no paper effect) when testing for nonzero variances. “Prop. sig.” and “Prop. sig. with RPE” are the proportion of randomization cases where the focal moderator was significant, without or with a RPE in the model, respectively (if there were no issues with the statistical model, such as uncorrected nonindependence, these proportions should be 0.05). *Abbreviations:* NR, not reported. NA, not applicable.

## RESULTS

### Effects of nonsense variables

**FIGURE 1:**
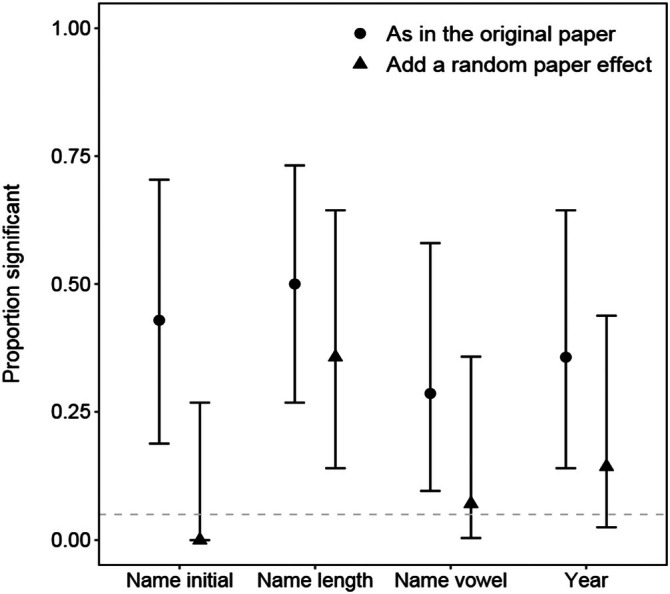
Proportion (with CIs) of 14 meta‐analyses (MAs) for which the effect of a nonsense variable concerning the author's name or publication year (see *Methods*) was significant at the *p* = 0.05 level without (blue, solid circle) and with (yellow‐orange, triangle) a random paper effect added to the analysis. CIs are Wald CIs using a variance based on a binomial distribution.

For all four nonsense variables, the distribution of the 14 *p*‐values deviated significantly from a uniform distribution (KS tests all *p* < 0.05). Indeed, across the four nonsense variables, from 4 to 7 of the 14 MAs (i.e., 28%–50%) found the nonsense variable to be significant, a result that substantially and significantly exceeded the null expectation of 0.7 MAs (i.e., 5% of the 14 MAs: Figure 1). Thus, for example, the length of the first author's name and whether a paper was published in an odd or even year had a significant effect 50% and 35% of the time, respectively. Adding a random paper effect reduced the number of MAs that detected a significant moderator effect to 0–5 (or 0%–36% of the 14 MAs), and while the proportion exceeded 0.05 in three of the four cases, the CIs overlapped 0.05 in three of the four cases. Thus, adding a random paper effect greatly reduced Type I error (falsely detecting a significant effect of a nonsense moderator), but it did not eliminate the problem.

### Effects of a random dummy variable

**FIGURE 2:**
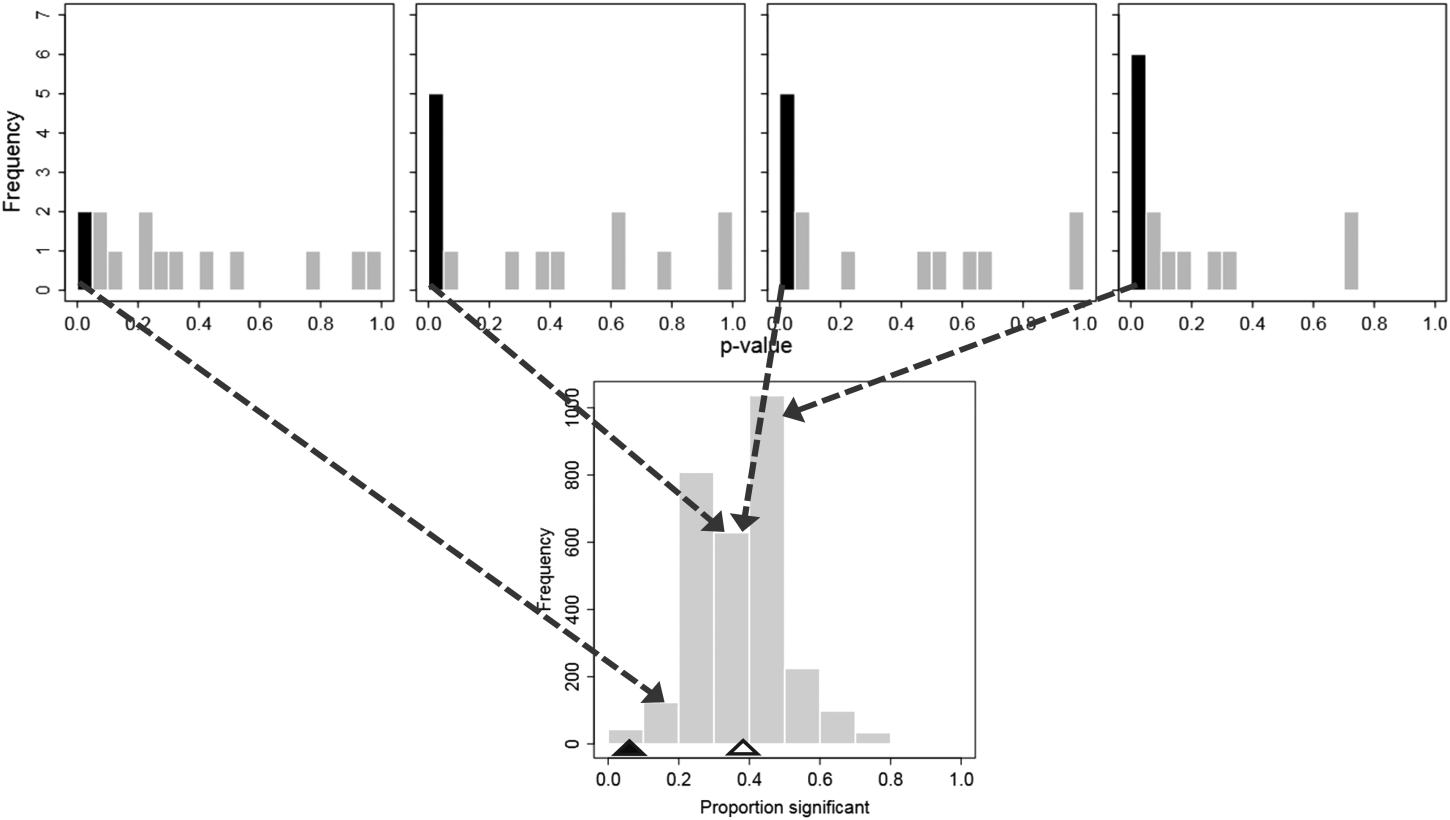
Frequency of *p*‐values (top row) and significant results (bottom panel) for the test of the dummy variables. Top: Four example (out of 3000) frequency distributions for realizations in the resampling analysis, with each example showing the distribution of *p*‐values for the 14 chosen meta‐analyses (MAs). Bottom: The frequency distribution (of the 3000 realizations) of the proportion of the 14 MAs that were significant in each realization. The first dark bar in each panel in the top row gives the frequency of significant results, and the dashed arrows from that bar to a bar in the bottom panel show which bar in the bottom panel the example in the top panel contributed to. The solid and open triangles on the *X*‐axis of the bottom panel demark the expected proportion (0.05) and the observed (average) proportion (0.38), respectively.

When we tested the effect of a random dummy variable (moderator), a majority of the 3000 distributions (75.5% [95% CI: 73.9%–77.0%]) deviated significantly from a uniform distribution, with an overrepresentation of *p*‐values below 0.05, as seen in three of the four examples illustrated (Figure 2 top). The average percentage of the 14 MAs in any given realization that declared the randomly assigned dummy variable significant (*p* < 0.05) was 38.2% (95% CI: 37.8%–38.7%; Figure 2 bottom), which exceeded the expected percentage of 5%. Thus, a high percentage of MAs using the same methodology and data as that in the originally published MA yielded a significant result when a random dummy variable was assigned to each group.

**FIGURE 3:**
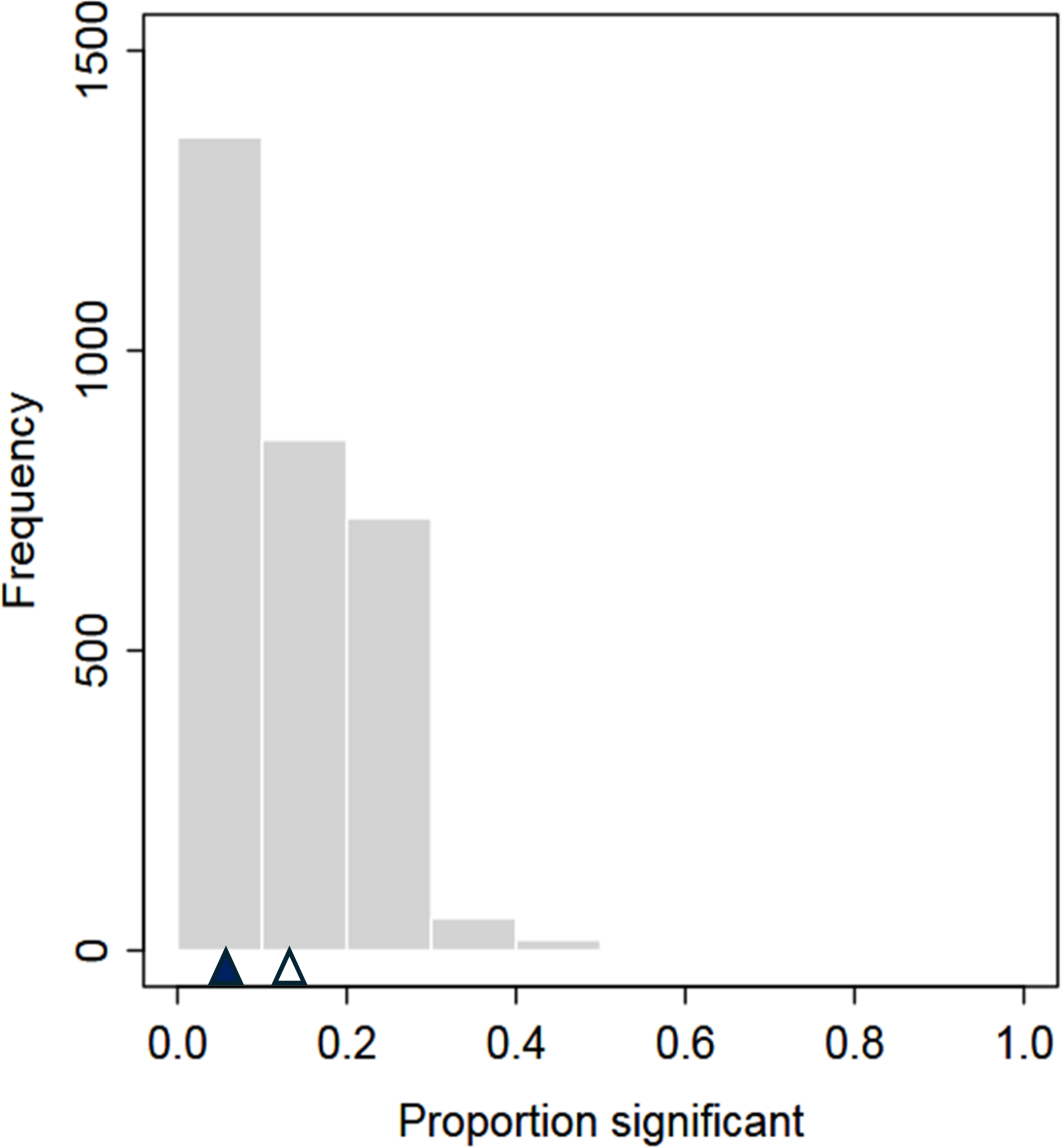
As in Figure 2 (bottom) but with a random paper effect. Frequency distribution over the 3000 realizations of the proportion of significant dummy variables for the 14 meta‐analyses (MAs) in each realization, when a random paper effect was added to the model. The solid and open triangles on the *X*‐axis demark the expected proportion (0.05) and the observed (average) proportion (0.127), respectively.

Again, including a random paper effect in the MA reduced, but did not eliminate, the problem of inflated significance (Figure 3): for example, we still observed significant results (i.e., *p* < 0.05), more frequently than 5% of the time. The percentage of realizations with significant departure from a uniform distribution was 16.3% (95% CI: 15.0%–17.7%), which although considerably lower than when we did not incorporate a random paper effect (75%), was still more than three times more common than expected (i.e., 5%). The average percentage of the 14 MAs in any given realization that were significant (*p* < 0.05) was reduced to 12.7% (95% CI: 12.4%–13.1%) from 38%.

**FIGURE 4:**
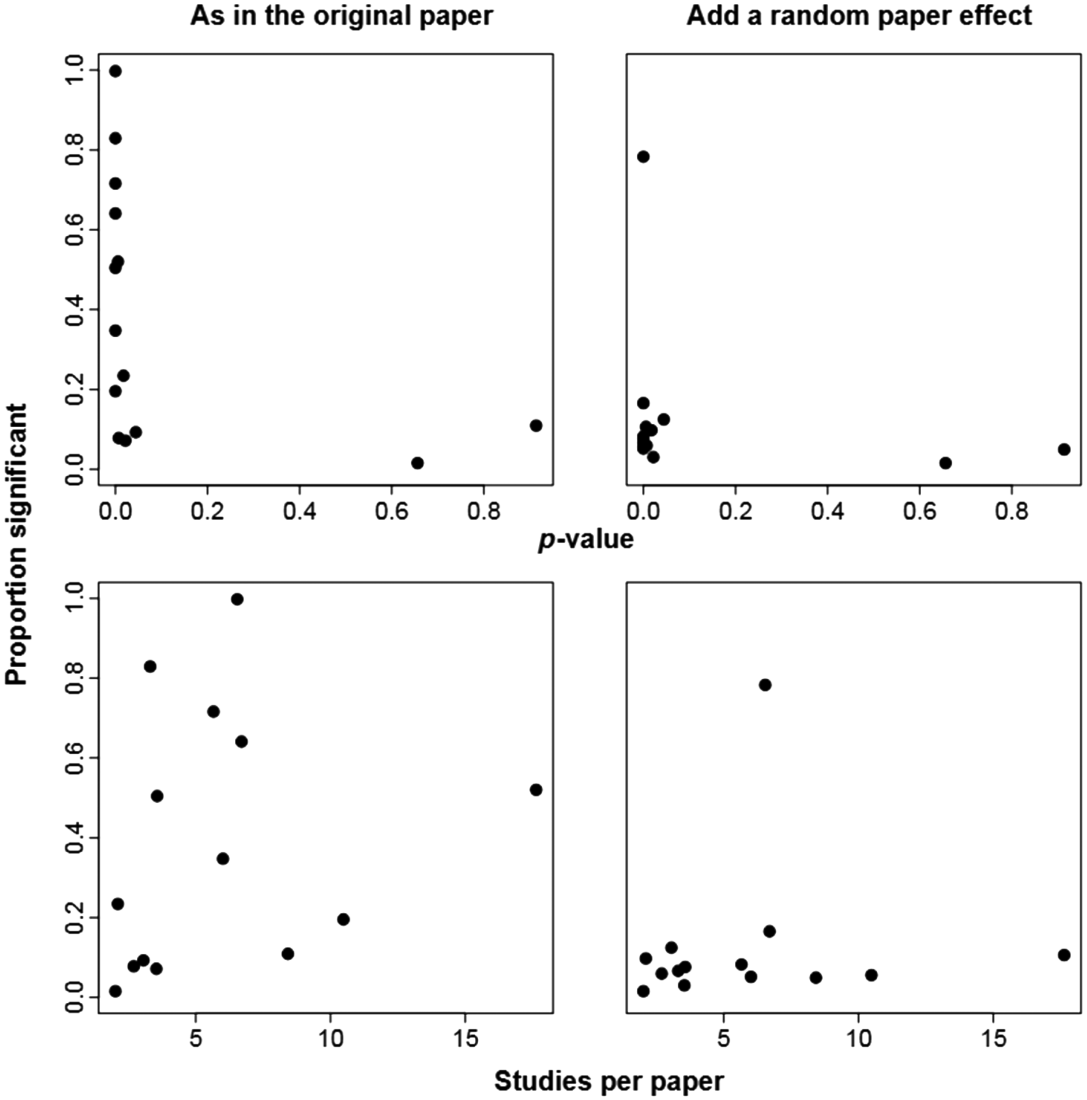
Proportions of cases where the dummy variable was significant calculated over all 3000 realizations for each of the 14 meta‐analyses. In the top row these are plotted versus the *p*‐value for the original meta‐analysis test, and in the bottom row they are plotted versus the average number of studies per source paper in each meta‐analysis. The left column gives the proportion significant using the model as reported in the original published meta‐analysis (without a random paper effect) and the right column gives the proportion significant for the modified model including a random paper effect.

A priori, we hypothesized that the propensity of a MA to yield false significant *p*‐values in tests of the dummy variable could be negatively related to the *p*‐value in the original analysis, given the same factors might influence both. In addition, we hypothesized that the propensity of false significant effects would be more pronounced if the papers in the MA included more observed effect sizes per paper, because the existence of larger groups should, all else being equal, lead to a larger influence of group nonindependence. We did not, however, see any significant relationships between the average proportion of significant effects and either of these variables, both in our repeat of the original analyses and after a random paper effect was added to those analyses (all *p* > 0.1, Figure 4). The influence of including a random paper effect is further seen in the generally lower proportion of times a particular MA had a significant dummy variable effect, when a random paper effect was included (Figure 4, compare right column to left).

### Effect of the random paper effect in the original analyses

In general, including a random paper effect led to large proportional increases in the *p*‐value of the original tests in the MA, with the increase being more than 30‐fold in 9 of 14 cases (Table 2). Of the 5 papers with a *p*‐value initially in the range of 0.001–0.05, 4 switched after incorporating a random paper effect from significant to nonsignificant (i.e., *p* > 0.05). That is, four out of those five papers would have yielded a qualitatively different inference had the authors accounted for nonindependence by including a random paper effect. Whereas some increase in *p*‐values with the addition of a random group effect is expected due to a loss of 1 df, and thus statistical power, this cost should be rather small. In addition, in 12 of the 14 cases and all 9 cases with a 30‐fold change in *p*‐values, the paper effect itself was significant (Table 2). Thus, results indicate a large influence of paper effects across the studies.

## DISCUSSION

Our analysis revealed that nonindependence introduces a profound effect on MA results, making the inferences of many studies that did not account for nonindependence problematic. We found that nonsense treatment variables assigned to a paper were significant far too often (e.g., 28%–50% instead of 5%). For example, assigning a moderator based on the percentage of vowels in the first author's name led to a significant effect of that moderator 50% of the time. Of course, any real effect of these nonsense variables is by design, nonsensical, but illustrates the problem in a way we have found to be intuitive and entertaining, and aids in drawing interest and concern from ecologists. More formal analyses using a dummy variable that was randomly assigned at the paper level, which allows statistical analysis of deviations from expectations, supports the results of the analyses based on nonsense variables. The dummy treatment variable was also significant too often (on average 38% instead of the expectation for no effect of 5%). Thus, the excess frequency of significant results must be tied to violations of the statistical assumptions of the MAs we evaluated. Because there should be no difference between the mean effect sizes between groups defined by the random assignment procedure, the results indicate that there was nonindependence within papers. One potential way to address the within‐paper nonindependence is to use a hierarchical model that explicitly incorporates a random paper effect (Nakagawa et al., 2017, 2022; Song et al., 2020). Indeed, when we modified the original analyses by incorporating random paper effects the percentage of randomizations that produced significant results was much reduced, from 38% to 16%. This percentage, however, remained substantially above the nominal level of 5%. This greater than 3‐fold inflation of Type I errors even after incorporating a random paper effect represents a serious statistical problem. Thus, simply incorporating random paper effects as a routine omnibus solution is not a sufficient solution to nonindependence.

Our study thus provides direct evidence supporting previous suggestions that unaccounted‐for nonindependence is a serious issue in MA (Nakagawa et al., 2017, 2022; Song et al., 2020). Our analyses, based on actual ecological MAs, rather than on simulations assuming nonindependence (as in Nakagawa et al., 2022; Song et al., 2020), show that nonindependence is sufficient to substantially distort ecological inferences. Our results also support the view that using hierarchical models, which incorporate random additive group effects (like a paper effect), is a powerful, but not necessarily sufficient, tool to address some forms of nonindependence (Nakagawa et al., 2017, Nakagawa et al., 2022; Song et al., 2020, 2022).

The excess of low *p*‐values that persisted even after we incorporated a paper effect suggests that the form of the paper effect causing issues was more complex in at least some of the MAs than assumed in the hierarchical model we applied, in which the random paper effect is normally distributed with all studies within a paper being equally correlated. More complex correlation structures might arise if there are other groupings in addition to paper (e.g., due to phylogenetic nonindependence). Thus, while a paper effect might capture some of the influences of other groupings (a single paper might focus on a single clade), it probably does not capture all aspects. The obvious, but not easy, solution is to identify the important groupings and include these in the analysis.

Another possibility is that observed effect sizes from a single paper might not be equally correlated (which is assumed by including paper as a random effect): for example, because some effect sizes in a paper shared a control while others did not, or because some effect sizes were based on different variables measured from the same experimental units, while others used different experimental units (Nakagawa et al., 2017). This problem can be addressed by incorporating a variance–covariance matrix describing the correlations among effects (Lajeunesse, 2011), or in some cases adopting a robust variance estimation approach (Hedges et al., 2010; Pustejovsky & Tipton, 2018). However, these approaches are sufficiently complex that they may be inaccessible to some analysts. In addition, while there are formulae to determine some forms of covariance, there are remaining challenges obtaining reliable empirical estimates of covariance in many circumstances. Another possibility is that the paper effect might be highly non‐normal so that sets of extreme observations common to a paper cannot be fully explained by normally distributed paper effects. This would be a particular issue when one or a few papers contribute a large number of consistently high (or low) observed effect sizes.

To some extent, Song et al. (2020) addressed the issue of unequal correlations in their simulations and found that incorporating an additive group effect was an effective solution. Their simulations, however, did not consider extreme cases in which the correlations were highly variable. We know of no evaluations of either non‐normal random group effects or highly variable correlation structures. We suspect that many issues might be ameliorated if the underlying groups or variables driving correlations were identified and incorporated into analyses, although our results reflect that including only a paper effect cannot fully resolve the issue. Thus, these issues require further research.

Thoughtful incorporation of potential correlation structures is required to perform a valid MA on ecological data (Lajuenesse, 2011; Nakagawa et al., 2017; Song et al., 2020; this study). Because most MAs extract >1 observed effect size from each primary paper (Pappalardo et al., 2023), one approach is to carefully consider the study design of source papers contributing more than one observed study, and to incorporate covariance structures for each paper based on the study design for that paper (Mengersen et al., 2013; Nakagawa et al., 2017; Noble et al., 2017). One might also more carefully examine the residuals and predicted random effects from a fitted model to more rigorously evaluate model adequacy. Residual analysis for models with random effects can be complex, but evolving tools such as so‐called one‐step‐ahead residuals (Thygesen et al., 2017) are becoming more accessible (e.g., implemented in the R package RTMB). Routine evaluation of whether results are sensitive to one or a small number of studies should be a routine part of evaluating the robustness of MAs.

Herein we evaluated the consequences of one general type of nonindependence, namely that due to a paper effect: that is, nonindependence in observed effect sizes taken from the same paper. In their review of ecological MAs Pappalardo et al. (2023) found that 95 out of 96 MAs included more effects than papers, with the most extreme MA including 52 papers that yielded a total of 46,347 effects! Pappalardo et al. (2023) provide evidence that as recently as 2019 only a small fraction of analyses acknowledged and accounted for nonindependence at the study level, a type of nonindependence one would expect when there are multiple observed effect sizes within groups (like papers). While we cannot refute the possibility that there has been a recent trend to better account for nonindependence, and in fact suspect this is true, our own exposure to the literature confirms that many studies still dismiss or ignore the issue.

Nonindependence from paper effects clearly underrepresents the general issue caused by nonindependence, as there will also be nonindependence among effect sizes taken from different papers. Indeed, phylogenetic MA methods were developed to account for correlated responses of closely related species, which is a factor that operates among as well as within papers (Adams, 2008; Chamberlain et al., 2012). Studies might more generally be classified by their similarity due to factors other than taxonomic relatedness of the studied species. One simple example is that observed effect sizes from multiple papers published by the same authors or by a related group of authors (some of the same ones, or authors with shared history) might be correlated because they used similar methods or were done in similar systems or geographic locations, hence having more similar true effect sizes. Similarly, a primary paper might also report somewhat redundant measures, such as growth rate and biomass of a target species, and it is not uncommon for these to be used to calculate multiple observed effect sizes. The general approach used in phylogenetic MAs might be adapted and have broader utility than is currently recognized. If the attributes associated with each effect size (e.g., location, investigator, organismal trait) could be used to define the “distance” between each effect, then this measure of distance could take the place of phylogenetic distance, and the basic tools used to incorporate phylogenetic correlations into MA could be applied more generally.

Historically, one reason nonindependence was not accounted for in past studies was likely due to the limited development of hierarchical meta‐analytic methods. For example, the most widely used software during the 1990s and 2000s was Metawin (Rosenberg et al., 2000), which did not allow for hierarchical models (because those methods had not yet been developed) nor did it discuss other ways to attempt to address nonindependence. Conceptual developments incorporating hierarchical random effects and other error structures for MA were slower than in the standard statistical arena, and thus software also lagged behind. Fortunately, the most widely used and currently available software for ecological MAs (the R package metafor: Viechtbauer, 2010) allows for hierarchical models and other correlated error structures, as does other software, including packages specifically developed for phylogenetic MA. However, despite the advent of these tools, and while we think it likely that there has been a recent trend to better account for nonindependence, many studies still dismiss or ignore the issue. Thus, there is an ongoing need for practitioners to consider the possible forms of nonindependence that might influence their MA data, and account for such correlations in their analyses. This might be easier said than done, and thus may require that many MA studies include a statistician experienced with MA in their team.

Our randomization approach relied on the expected distribution of *p*‐values from repeated sampling. Therefore, it does not allow for rigorous evaluation of whether a specific MA is problematic. However, we think there is some potential to use a simulation approach to identify potential problems with a particular individual analysis, and this would provide a way to evaluate if changes made to address potential within‐paper nonindependence have adequately addressed the problem. The meta‐analyst will know the details of the design (how many papers, how many observed effect sizes for each paper, etc.) and can simulate multiple meta‐datasets based on these details and the underlying statistical assumptions (and parameters estimated when fitting the model). The analyst could then evaluate, based on the simulations, what proportion of random assignments to a dummy variable led to a significant dummy variable effect. The distribution of these proportions could be used as a null distribution for the proportion found significant by randomization using the actual data. If the observed proportion from randomization was high relative to the null distribution, this would indicate the specific MA remained problematic, whereas if the randomization produced similar proportions that were significant as in the simulations, this would suggest an adequate solution had been found.

In our research and interactions with colleagues, we have found the use of nonsense variables to be a valuable means of communicating the problem posed by nonindependence— showing that a meaningless variable creates strong and spurious results resonates with most ecologists. We are not the first to have used this device. For example, pixel color from classic paintings overlain on species distribution maps predicted species distributions as well as many of the best environmental models (Fourcade et al., 2018). As is the case with nonindependence in MA, the issues causing the problems with species distribution models were already well established, but showing intuitively that they mattered for real analyses had a profound effect. Similarly, evaluating fitted models using randomization has been widely applied in other contexts: for example, in specifying ecological null models (Gotelli & Urlich, 2012). As another example, random assignment of species to functional groups has been used to evaluate the effects of functional group diversity on ecosystem processes, and this has shown that standard parametric evaluations that assume known functional groups can be misleading (Petchey, 2004; Wright et al., 2006). As we found in our research, specifying appropriate null models and randomization can be tricky, and simulations can prove a useful tool to check what seems to be logical and sound thinking (Gotelli & Urlich, 2012).

We have demonstrated that unaccounted‐for nonindependence due to paper effects often has a large and significant effect on the interpretation of ecological MAs. Our focus has been on the need to account for paper effects as well as other sources of nonindependence to best examine the role of ecological moderators. However, we caution that presumed nonindependence might arise for sound ecological reasons. For example, if ecological venue (e.g., size of experimental units, or lab vs. field experiment) affects the outcome of studies, and venue is similar within a group (e.g., a paper reports multiple studies using the same venue), and the meta‐analyst does not incorporate venue as a moderator, the role of this hidden moderator will appear as a “group effect.” Thus, if correlated effect sizes are observed within groups (e.g., via a paper effect), then investigators should clearly think about what might be causing this similarity within groups and if potential explanatory moderators might resolve this effect. Such an approach would pay dividends in two ways. First, it would reduce the magnitude of nonindependence by explaining some of the shared variation, thus increasing power to detect the influences of moderators that were originally of interest. Second, it would improve ecological understanding by identifying additional factors influencing a process. That said, ecological systems and studies are unavoidably complex and heterogeneous, so we suspect there will typically be unknown factors causing similarities in observed effect sizes within groups despite best efforts to ferret them out.

In conclusion, synthesis plays a large role in the field of ecology (Pappalardo et al., 2023), as it does across the sciences (Gurevitch et al., 2018; Hoffmann et al., 2021; Vrieze, 2018). MA was proposed as a more rigorous approach focused on observed effect sizes and exploring causes for variation in those effects. However, nonindependence remains an issue in ecological MA (O'Dea et al., 2021; Pappalardo et al., 2023). We agree with Nakagawa et al. (2017), who argued that results from studies (in the published literature or in the review process) that appear to ignore or glibly dismiss potential issues of nonindependence should be viewed critically. While it may not be possible to reanalyze past MA publications using more appropriate methods, previously published MAs that did not account for or evaluate nonindependence are likely to have overstated the strength of evidence supporting their conclusions. Nonindependence among observed effect sizes may be more pervasive in ecological MAs than in MAs in some other fields because of the large size and complex data structure of many ecological MAs, and the large number of ways in which nonindependence can arise. However, nonindependence is a ubiquitous problem for research synthesis in most research fields, and much work remains to be done to better model and account for sources of nonindependence. More generally, it is incumbent on analysts to consider plausible violations of assumptions, and in the face of evidence for such violations, the analysts should adjust procedures. However, there is no guarantee the adjustment will remove all issues with nonindependence—as our evaluation of including random paper effects demonstrate—and available diagnostics and procedures will continue to evolve. One might only move forward to publish an analysis if the potential statistical issues can be convincingly argued to be unlikely to undermine the key results, and even so, conclusions need to be constrained by carefully articulated caveats. This also supports the increasing calls for reproducible analyses and accessible data so that data can be re‐analyzed as new tools and approaches become available. This is true not only for primary studies but also in MAs.

## AUTHOR CONTRIBUTIONS

James Bence, Craig Osenberg, Scott Peacor, and Chao Song designed the statistical approach and the simulations. Chao Song built the model and performed simulations. Amy Briggs, Elizabeth Hamman, and Scott Peacor gathered and assembled the data. James Bence and Scott Peacor wrote the first draft of the manuscript, and all other authors contributed significantly to its content.

## CONFLICT OF INTEREST STATEMENT

The authors declare no conflicts of interest.

## Supporting information


Appendix S1.

## Data Availability

Data and code (Peacor et al., 2025) are available in Figshare at https://doi.org/10.6084/m9.figshare.28187996.
