# Supplementary material for: Ecological meta‐analyses often produce unwarranted results
Source: Ecology. 2025 Dec 8;106(12):e70269. doi: 10.1002/ecy.70269 (PMC12683612; doi:10.1002/ecy.70269)
Supplement: Supplementary file 1 — Appendix S1. [file ECY-106-e70269-s001.pdf]

APPENDIX S1

Journal:

Ecology

Title:

Ecological meta-analyses often produce unwarranted results

Authors:

Scott D. Peacor, Chao Song, James R. Bence, Amy A. Briggs, Elizabeth A. Hamman, Craig W. Osenberg

Contents:

Figure S1. Prisma diagram

Table S1. Paper IDs and full citations for the 20 selected meta-analyses

Figure S1. Prisma diagram

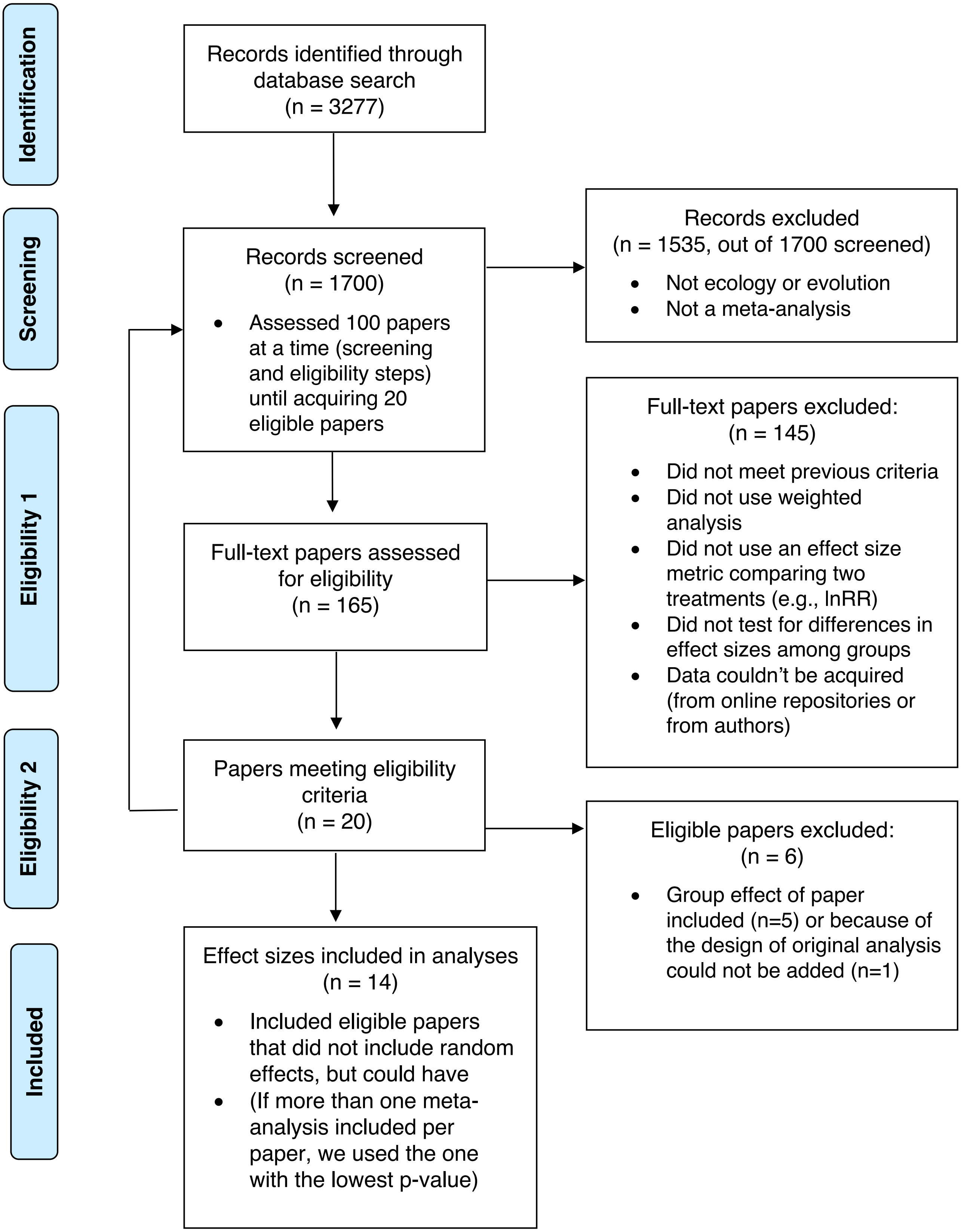

Table S1. Paper IDs and full citations for the 20 selected meta-analyses.

Paper ID	Citation
2	Shimamoto, C.Y., Padial, A.A., da Rosa, C.M. & Marques, M.C.M. (2018). Restoration of ecosystem services in tropical forests: a global meta-analysis. PLoS ONE, 13, e0208523.
53	Yan, G.Y., Mu, C.C., Xing, Y.J. & Wang, Q.G. (2018). Responses and mechanisms of soil greenhouse gas fluxes to changes in precipitation intensity and duration: a meta-analysis for a global perspective. Canadian Journal of Soil Science, 98, 591–603.
82	Ouyang, Y., Evans, S.E., Friesen, M.L. & Tiemann, L.K. (2018). Effect of nitrogen fertilization on the abundance of nitrogen cycling genes in agricultural soils: a meta-analysis of field studies. Soil Biology and Biochemisty, 127, 71–78.
120	Zhang, L.Y., Jing, Y.M., Xiang, Y.Z., Zhang, R.D. & Lu, H.B. (2018). Responses of soil microbial community structure changes and activities to biochar addition: a meta-analysis. Science of the Total Environment, 643, 926–935.
272	Davidson, I.C., Cott, G.M., Devaney, J.L. & Simkanin, C. (2018). Differential effects of biological invasions on coastal blue carbon: a global review and meta-analysis. Global Change Biology, 24, 5218–5230.
282	Moreira, X., Abdala-Roberts, L. & Castagneyrol, B. (2018). Interactions between plant defence signalling pathways: evidence from bioassays with insect herbivores and plant pathogens. Journal of Ecology, 106, 2353–2364.
303	Santana, M.S., Sandrini-Neto, L., Neto, F.F., Ribeiro, C.A.O., Di Domenico, M. & Prodocimo, M.M. (2018). Biomarker responses in fish exposed to polycyclic aromatic hydrocarbons (PAHs): systematic review and meta-analysis. Environmental Pollution, 242, 449–461.
424	Cooper, E.B. & Kruuk, L.E.B. (2018). Ageing with a silver-spoon: a meta-analysis of the effect of developmental environment on senescence. Evolution Letters, 2, 460–471.
511	Monsreal-Ceballos, R.J., Ruiz-Sánchez, E., Ballina-Gómez, H.S., Reyes-Ramírez, A. & González-Moreno, A. (2018). Effects of botanical insecticides on Hymenopteran parasitoids: a meta-analysis approach. Neotropical Entomology, 47, 681–688.
585	Zhou, Z.H., Wang, C.K. & Luo, Y.Q. (2018). Response of soil microbial communities to altered precipitation: a global synthesis. Global Ecology and Biogeography, 27, 1121–1136.
628	Bugnot, A.B., Mayer-Pinto, M., Johnston, E.L., Schaefer, N. & Dafforn, K.A. (2018). Learning from nature to enhance blue engineering of marine infrastructure. Ecological Engineering, 120, 611–621.
639	de Lima, D.O., Lorini, M.L. & Vieira, M. V. (2018). Conservation of grasslands and savannas: A meta-analysis on mammalian responses to anthropogenic disturbance. Journal for Nature Conservation, 45, 72–78.

674	Zupan, M., Fragkopoulou, E., Claudet, J., Erzini, K., Costa, B.H.E. & Gonçalves, E.J. (2018). Marine partially protected areas: drivers of ecological effectiveness. Frontiers in Ecology and the Environment, 16, 381–387.
695	Alster, C.J., Weller, Z.D. & von Fischer, J.C. (2018). A meta-analysis of temperature sensitivity as a microbial trait. Global Change Biology, 24, 4211–4224.
759	Banks, P.B., Carthey, A.J.R. & Bytheway, J.P. (2018). Australian native mammals recognize and respond to alien predators: a meta-analysis. Proceedings of the Royal Society B-Biological Sciences, 285, 20180857.
952	Lekberg, Y., Bever, J.D., Bunn, R.A., Callaway, R.M., Hart, M.M., Kivlin, S.N., et al. (2018). Relative importance of competition and plant-soil feedback, their synergy, context dependency and implications for coexistence. Ecology Letters, 21, 1268–1281.
1180	Koricheva, J. & Hayes, D. (2018). The relative importance of plant intraspecific diversity in structuring arthropod communities: A meta-analysis. Functional Ecology, 32, 1704–1717.
1185	Sciberras, M., Hiddink, J.G., Jennings, S., Szostek, C.L., Hughes, K.M., Kneafsey, B., et al. (2018). Response of benthic fauna to experimental bottom fishing: A global meta-analysis. Fish and Fisheries, 19, 698–715.
1307	Dong, Y.L., Wang, Z.Y., Sun, H., Yang, W.C. & Xu, H. (2018). The response patterns of arbuscular mycorrhizal and ectomycorrhizal symbionts under elevated CO2: A meta-analysis. Frontiers in Microbiology, 9, 1248.
1365	Mandich, M. (2018). Ranked effects of heavy metals on marine bivalves in laboratory mesocosms: A meta-analysis. Marine Pollution Bulletin, 131, 773–781.
